# Antegrade urethrogram: A technique to visualize the proximal bulbous urethral segment in anterior urethral stricture

**DOI:** 10.4103/0970-1591.56193

**Published:** 2009

**Authors:** Apul Goel, Ankush Gupta, Divakar Dalela

**Affiliations:** Department of Urology, King George Medical University, Lucknow – 226 003, Uttar Pradesh, India

**Keywords:** Stricture urethra, urethrogram, voiding cystourethrography

## Abstract

In patients of stricture urethra that are on suprapubic catheter if the proximal bulbous urethral segment is not visualized at the time of voiding cystourethrography, antegrade urethrogram can be done. Through the suprapubic tract ureteric catheter is passed cystoscopically into the proximal urethra and contrast instilled to visualize the proximal urethra.

## INTRODUCTION

A combination of retrograde urethrogram (RGU) and voiding cystourethrogram (VCUG) is done to define the site and length of urethral stricture. Sometimes, at the time of VCUG the bladder neck does not open leading to overestimation of stricture length.[1,2] Another factor that can lead to overestimation of stricture length is that many patients are on suprapubic diversion for a long time that leads to small bladder capacity. Instilling contrast in the bladder causes discomfort with resultant incomplete bladder distention sufficient to open bladder neck voluntarily.[3] Magnetic resonance imaging is another method to noninvasively assess the length of stricture.[4] However, it is difficult to interpret by urologists.

As many patients of urethral stricture are on a suprapubic catheter, we describe a simple method to visualize the proximal bulbous urethral segment in such cases. Dalela *et al.* described a similar method where the patient had formed synechia in proximal urethra due to prolonged urinary diversion that did not allow proper filling of the proximal urethral segment. Antegrade cystoscopy was done to break the synechia and antegrade urethrogram was performed.[5]

## TECHNIQUE

**Figure 1:**
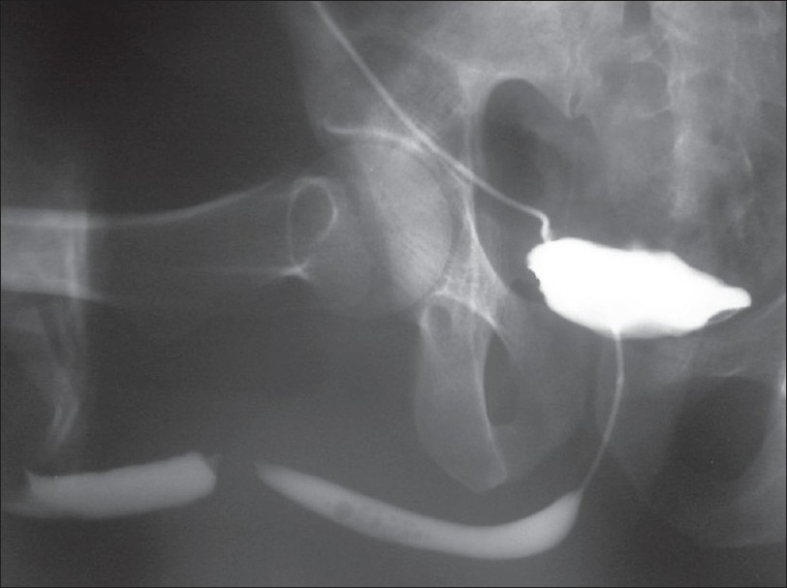
Combined antegrade and retrograde urethrogram in a patient with penobulbous junction stricture. Retrograde urethrogram shows complete obliteration of urethral lumen at the penobulbar junction. Antegrade urethrogram delineates the proximal urethral segment till the strictured area

In patients of stricture urethra with suprapubic catheter and where the proximal bulbous urethra is not visualized on VCUG, antegrade urethrogram using our technique was performed. Antegrade cystoscopy is performed through the suprapubic tract using a 19-F sheath and a 0° or 30° cystoscope lens. The cystoscope is maneuvered into the bladder neck and a 5-F ureteric catheter passed into the proximal urethra. This can be performed using the flexible endoscope also. Radiocontrast agent is then pushed through the ureteric catheter to visualize the proximal urethral segment either on C-arm fluoroscopy or on static radiographic films [Figure 1]. We have used this technique in five cases with excellent results.

## DISCUSSION

Antegrade urethrogram is an accurate method to visualize the proximal bulbous urethral segment in patients who are already on a suprapubic catheter. It is done just before the planned urethroplasty under anesthesia. Sometimes, additional procedures like removal of small bladder stones can also be performed simultaneously. To delineate the proximal urethral segment antegrade passage of curved metal sound into the posterior urethra has been described for patients with posterior urethral disruption.[4] However, it is painful and there is chance of injury to the bladder neck while introducing the metallic sound blindly.

**Figure 2:**
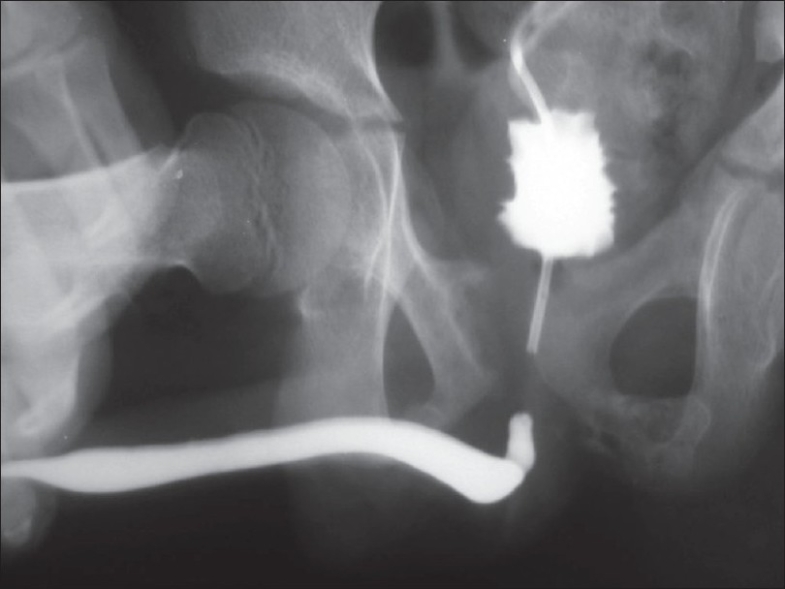
Combined antegrade and retrograde urethrogram in a patient with posterior urethral distraction injury. On pushing contrast, the contrast immediately enters the bladder without filling the proximal urethral segment

No method to visualize the proximal anterior urethra is described when it is not visualized on VCUG. Antegrade urethrogram, although required rarely, is a very useful technique in such situations. It is not a good technique to visualize the prostatic urethra in cases of posterior urethral disruption as it is difficult to maintain the ureteric catheter in the posterior urethra and the contrast rapidly enters the bladder without properly filling the posterior urethra [Figure 2]. A disadvantage of the procedure is that urological expertise is needed for this investigation. Percutaneous antegrade urethrogram as part of treatment of urethral stricture under radiographic control has also been described.[6,7] However, the technique is cumbersome and epidural anesthesia is needed in most cases.[7]
